# Insights Into a *Chlamydia pneumoniae*-Specific Gene Cluster of Membrane Binding Proteins

**DOI:** 10.3389/fcimb.2020.565808

**Published:** 2020-10-21

**Authors:** Corinna Braun, Johannes H. Hegemann, Katja Mölleken

**Affiliations:** Institute of Functional Microbial Genomics, Heinrich-Heine-University, Düsseldorf, Germany

**Keywords:** *Chlamydia pneumoniae*, membrane binding proteins, species specificity, DUF domain containing protein family, gene cluster

## Abstract

*Chlamydia pneumoniae* is an obligate intracellular pathogen that causes diseases of the upper and lower respiratory tract and is linked to a number of severe and chronic conditions. Here, we describe a large, *C. pneumoniae*-specific cluster of 13 genes (termed *mbp1-13*) that encode highly homologous chlamydial proteins sharing the capacity to bind to membranes. The gene cluster is localized on the chromosome between the highly diverse adhesin-encoding *pmp* genes *pmp15* and *pmp14*. Comparison of human clinical isolates to the predicted ancestral koala isolate indicates that the cluster was acquired in the ancestor and was adapted / modified during evolution. SNPs and IN/DELs within the cluster are specific to isolates taken from different human tissues and show an ongoing adaptation. Most of the cluster proteins harbor one or two domains of unknown function (DUF575 and DUF562). During ectopic expression in human cells these DUF domains are crucial for the association of cluster proteins to the endo-membrane system. Especially DUF575 which harbors a predicted transmembrane domain is important for binding to the membrane, while presence of the DUF562 seems to be of regulatory function. For Mbp1, founding member of the cluster that exhibits a very limited sequence identity to the human Rab36 protein, we found a specific binding to vesicles carrying the early endosomal marker PtdIns(3)P and the endosomal Rab GTPases Rab11 and Rab14. This binding is dependent on a predicted transmembrane domain with an α-helical / β-strand secondary structure, as the mutant version Mbp1mut, which lacks the β-strand secondary structure, shows a reduced association to PtdIns(3)P-positive membranes carrying Rab11 and Rab14. Furthermore, we could not only show that Mbp1 associates with Rab36, but found this specific Rab protein to be recruited to the early *C. pneumoniae* inclusion. Detection of endogenous Mbp1 and Mbp4 reveal a colocalization to the chlamydial outer membrane protein Momp on EBs. The same colocalization pattern with Momp was observed when we ectopically expressed Mbp4 in *C. trachomatis*. Thus, we identified a *C. pneumoniae*-specific cluster of 13 membrane binding proteins (Mbps) localizing to the bacterial outer membrane system.

## Introduction

*Chlamydia pneumoniae* (*Cpn*) is one of the two major pathogenic species of the Gram-negative *Chlamydiaceae* family of bacteria that infect humans, and is responsible for a variety of acute and chronic diseases of the upper and lower respiratory tract, such as pneumonia, asthma, and bronchitis (Hahn et al., 1991). All *Chlamydiae* are obligate intracellular parasites with a unique biphasic life cycle consisting of the alternation of two morphological forms: the infectious elementary body (EB), and the metabolically active, non-infectious reticulate body (RB) which replicates in the host cell (Chi et al., 1987; Miyashita et al., 1993; Wolf et al., 2000). Adhesion of EBs to their target cells is the first essential step in the infection process. This is followed by internalization of EBs into a membrane-bound compartment, termed the inclusion, in which EBs develop into replicative RBs. Initial contact between the *Cpn* EB and the host cell is mediated by binding of conserved adhesins to various host cell structures. Of these, OmcB binds to heparan-sulfate-like proteoglycans (GAG), while LipP (CPn0473) binds to phospholipids in the host membrane, and Pmp21 interacts with the epidermal growth factor receptor (EGFR), serving as both an adhesin and an invasin (Moelleken and Hegemann, 2008; Mölleken et al., 2013; Fechtner et al., 2016). This last interaction promotes internalization of the EB by activating the EGFR, and the developing inclusion at first remains associated with the activated receptor (Mölleken et al., 2013). Supported is the adhesin-receptor mediated internalization by type III (T3) secreted effector proteins. CPn0572 (TarP ortholog) binds actin and enhances actin polymerization at the entry site (Clifton et al., 2004; Jewett et al., 2010; Zrieq et al., 2017) while the most recently discovered SemC (CPn0678), binds and deforms the host plasma membrane (PM). SemC recruits the endocytic scaffold protein SNX9 to facilitate uptake of the EB into the cell (Hansch et al., 2020). During endocytosis of EGFR the internalized receptor is generally delivered to the early or sorting endosomal compartment, so that it is either recycled back to the PM or delivered to the lysosome for degradation, respectively (Madshus and Stang, 2009). However, *Cpn* disrupts these pathways to avoid both EGFR-triggered degradation and immediate recycling to the PM. Recently, we have shown that within an hour of initial EGFR-mediated adhesion, the nascent inclusion specifically acquires an early endosomal identity with a PtdIns(3)P-positive membrane (Molleken and Hegemann, 2017). Furthermore, several endosomal Rab GTPases are recruited to the early inclusion immediately after internalization. These include Rab4 and Rab11, both of which regulate fast and slow recycling (Lindsay et al., 2002; Campa et al., 2018), the late endosomal Rab7 (Stroupe, 2018), and Rab14, which is involved in the biosynthetic/recycling pathway between the Golgi and endosomal compartments (Junutula et al., 2004). While Rab11 and Rab14 remain associated with the inclusion membrane, Rab4 and Rab7 disappear 30 minutes after internalization (Molleken and Hegemann, 2017). Taken together, these findings suggest that *Cpn* actively regulates the membrane identity of the nascent inclusion by acquiring a specific lipid composition, and by avoiding degradation through the lysosomal pathway. This requires interaction of secreted bacterial virulence factors, so-called effector proteins, which either alter membrane lipid composition or recruit or displace Rab proteins or their modulating effectors. This is a conserved trait of intracellular pathogens that subvert host defense mechanisms and engage host organelles to establish their unique intracellular niches (Stein et al., 2012; Spano and Galan, 2018). Thus far many of those bacterial effectors share the common ability to interact with membrane structures of the host, by carrying membrane-binding domains (Weigele et al., 2017). Other effectors directly target host Rab GTPases in order to manipulate vesicular trafficking (Stein et al., 2012).

In this report, we analyzed the function of the newly identified gene cluster *GiD_A_04840-04720*, whose products are chlamydial membrane binding proteins. Proteins carrying the cluster-specific DUF575 domain are able to bind to different host endo-membranes during ectopic expression. Mpb1 localization to early endosomes carrying Rab GTPases, also found on the early inclusions, is dependent on a β-sheet sequence within the DUF575. During infection the two cluster proteins Mbp1 and Mbp4 colocalize with the outer membrane protein Momp on EBs and RBs.

## Results

### The Novel *GiD_A_04840-04720* Gene Cluster Is Specific for *Cpn*

**Figure 1:**
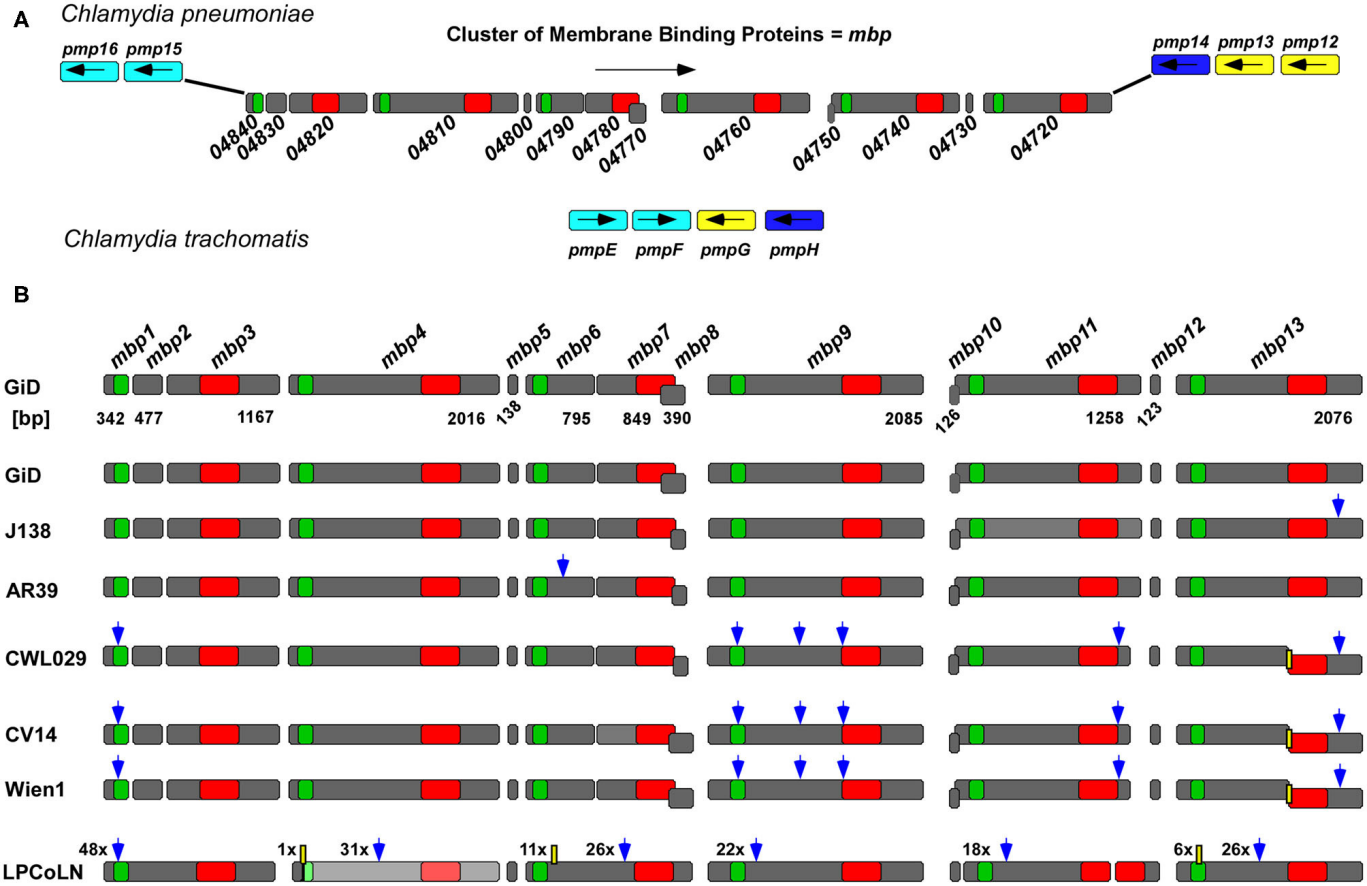
The *GiD_A_mbp1-mbp13* gene cluster is specific for *Cpn*. **(A)** Genomic context of the *Cpn* GiD *mbp1-mbp13* gene cluster located between *pmp15* (*C. tr*. homolog *pmpE/F*, light blue) and *pmp14* (*C. tr*. homolog *pmpH*, dark blue) in comparison to the configuration of the corresponding homolog genes in the genomic locus in *C. trachomatis* where *pmpG* (yellow) is inserted in between *pmpE/F* and *pmpH*. Each dark gray box symbolizes one gene, and the arrows indicate their orientations in the genome. The distribution of the two predicted DUF domains, DUF575 (green box) and DUF562 (red box) is indicated. **(B)** Comparison of the GiD *mbp1-mbp13* gene cluster with its counterparts from six different *Cpn* isolates from humans and the chlamydial strain LPCoLN recovered from the koala (*Phascolarctos cinereus*), which is regarded as a hypothetical ancestor of human *Cpn* strains. The scheme shows all detected sequence variations between the genes in different isolates relative to GiD. Blue arrow: bp exchange; yellow box: deletions (the digits give the number of exchanges or deletions). While GiD J138 and AR39 contain 13 genes in CWL029, CV14, and Wien1 *mbp13* is split into two genes due to a 1 bp deletion resulting in a premature stop and a new downstream start codon. In the LPCoLN isolate the *mbp4* homolog, indicated in light gray, is non-functional, carrying an N-terminal stop codon.

To identify chlamydial proteins with similarities to proteins involved in endocytic processes, we performed a bioinformatic screen of all 423 hypothetical proteins encoded by the genome of the *Cpn* GiD isolate (Jantos et al., 1997; Weinmaier et al., 2015) against the human genome, and sequences of effector proteins from various obligate intracellular bacterial pathogens. In this screen, the hypothetical protein GiD_A_04840 was identified, based on its 27.9% overall identity to the human GTPase Rab36, which is known to be involved in regulating vesicle traffic of late endosomes and lysosomes in the perinuclear region (Figure S1A) (Chen et al., 2010). However, a detailed bioinformatic analysis found no evidence that GiD_A_04840 acts as a Rab protein or a Rab mimetic, but revealed, that it belongs to a gene family consisting of 13 hypothetical genes, which code for proteins that display up to 59% pairwise sequence identity and which we termed “Membrane binding proteins 1-13” (Mbp1-13) (Figures 1A,B, Figure S1B). The size of the genes within the cluster differ significantly, with three genes being less than 140 bp long, while the others range from 342 to 2,085 bp in length. In our subsequent experimental studies, we focused on the products of the latter group.

The gene cluster is located between the adhesion relevant genes *pmp15* (*Ctr pmpE/F* and *pmp14* (*Ctr pmpH*), and is oriented in the opposite direction to these (Figure 1A). Interestingly, two of the small genes, *mbp8* and *mbp10*, are in a different reading frame from the others in the cluster. The 13 proteins encoded by the GiD gene cluster show an overall identity of up to 58.5% (Mbp4/ Mbp7) and an average identity of 32.6%. Moreover, eight of them harbor either one or both of the domains of unknown function, DUF575 and DUF562 (Figure 1B, Figure S1B). The DUF575 domain, with ~100 amino acids (aa) length, is highly conserved within the predicted products of the cluster, with pairwise identities ranging between 40.2% (Mbp6/ Mbp1) and 59.8% (Mbp11/ Mbp9) (Figure S1C). Alignments of DUF562, a domain of ~140 aa, show more variability in the aa sequences (Figure S1D), with pairwise comparisons yielding identity scores of between 24.6% (Mbp9/ Mbp3) and 66.2% (Mbp7/ Mbp4).

Sequence comparisons with the genomes of other *Chlamydiae* species showed that the gene cluster is present only in human *Cpn* strains and in the related, phylogenetically basal isolate LPCoLN from the koala (Figures 1A,B). Interestingly, detailed comparison of the GiD genes, which we used as reference strain, with those of five other human *Cpn* isolates and the ancestral koala isolate LPCoLN revealed significant differences (Figure 1B, Figures S2A,B). Compared to GiD, the LPCoLN gene cluster contains 6 larger genes each harboring an N-terminal DUF575 and a C-terminal DUF562. One of them (CPK0977) contains an N-terminal stop-codon and is therefore not expressed, while one (CPK0971) harbors a premature stop-codon leading to a truncated protein with an incomplete DUF562 domain. Interestingly, the remaining sequence of this DUF domain is found in the neighboring smaller gene (CPK0970). Additionally, the LPCoLN cluster carries only two small genes with <140 bp. Comparison of the GiD gene cluster with that from LPCoLN shows a large number of single nucleotide polymorphisms (SNPs), one 6-base-pair (bp) insertion (CPK0969), and two deletions [CPK0947 (11 bp) and CPK0977 (2 bp)] (Figure 1B, Figure S2B).

As the koala strain is discussed to resemble the hypothetical common ancestor of animal and human *Cpn* strains (Myers et al., 2009), the cluster comprising six genes with both DUF domains may be acquired by the ancestor of the koala isolate and LPCoLN exhibits the first signs of gene fragmentation (Figure 1B). In the younger human isolates, even more genes are found to be split, leading to the 13 ORFs seen today. Interestingly, the disposition and sizes of the two DUF domains are conserved among all isolates analyzed (Figure 1B, Figure S2A).

Among the human isolates the variations involve both SNPs as well as IN/DELs (insertions/ deletions) (Figure 1B, Figure S2B). One of the most interesting examples is a 1-bp deletion in the *mbp13* homologs in the strains CWL029, CV14, and Wien1, which results in a premature stop codon and a new downstream start codon. Thus, each of these isolates harbors two shorter genes with separated DUF domains instead of the long gene found in the GiD isolate (Figure 1B). When we compared the patterns of SNPs, deletions and insertions between GiD and the other respiratory (CWL029, AR39, J138) and vascular isolates (CV14, Wien1), we found that the coding sequences in GiD were virtually identical to those in AR39 and J138, with only one missense SNP distinguishing the three. Surprisingly, in this comparison the gene cluster from CWL029, a respiratory isolate, showed the same pattern of substitutions as the ones observed in the two vascular isolates (Figure 1B). Our small comparison is supported by the comparative analysis of 24 different *Cpn* isolates, which showed that genes of the cluster have about the same amount of SNPs as the highly diverse *pmp* genes (Weinmaier et al., 2015).

Taken together, this bioinformatic analysis characterizes a novel *Cpn*-specific gene cluster, which is conserved from the ancestral *Cpn* koala isolate to the modern human clinical strains. The distribution of mutations and IN/DELs among the strains leads to differences in terms of gene number and size within the cluster and probably represents an adaptation to host and/ or tissue.

### The DUF575 Enables Cluster Proteins to Bind to Host Membranes

**Figure 2:**
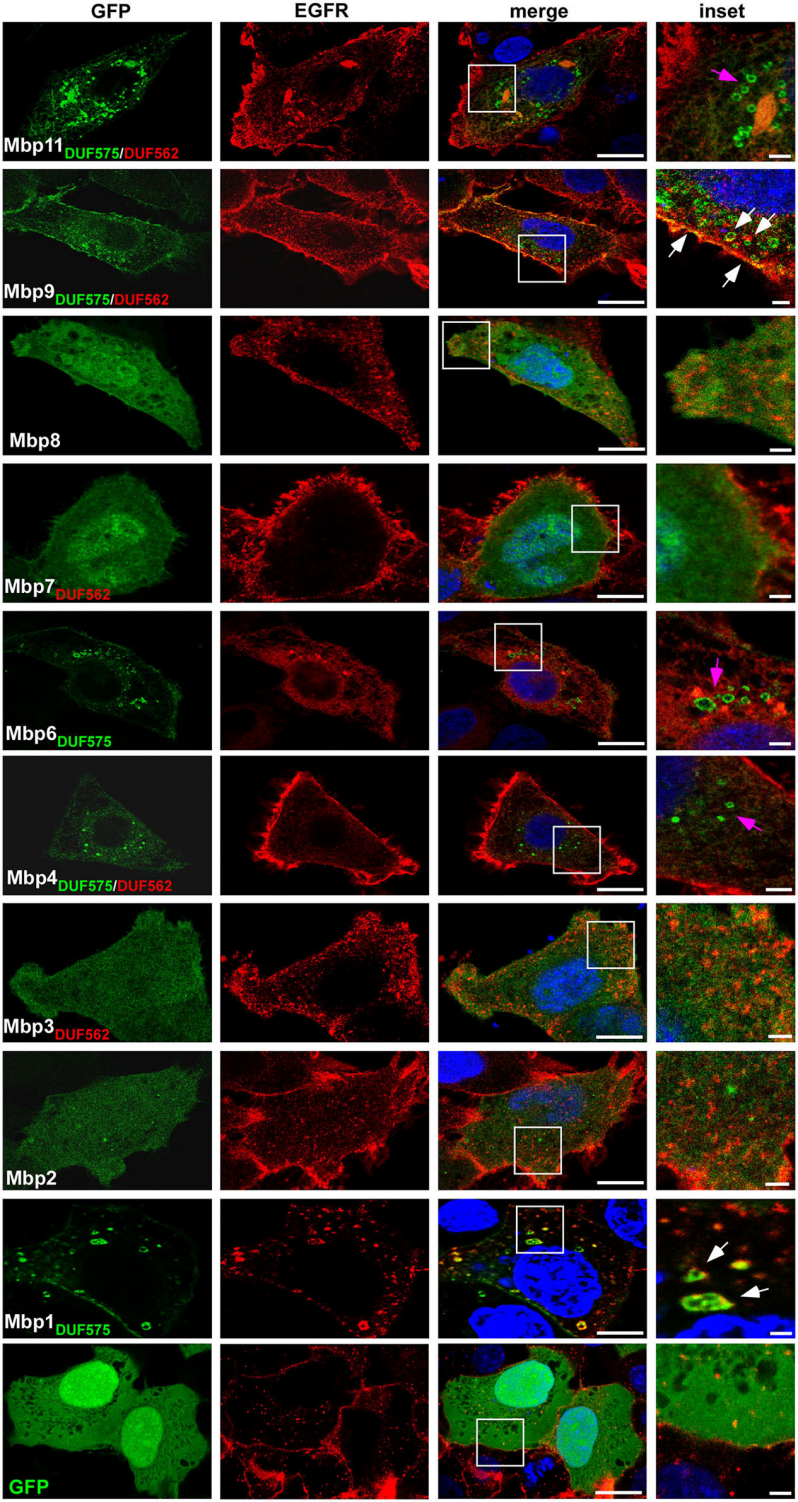
Ectopic expression of cluster proteins reveal a DUF575-dependent localization to EGFR-positive endo-membranes in human cells. Confocal images of human epithelial cells transfected with GFP or nine predicted products of the GiD gene cluster *mbp1*-*mbp11*, each fused to GFP. Presence of the DUF domains are indicated in green and red. HEp-2 cells were transfected with the indicated GFP fusion proteins for 18 h, fixed with 3% PFA and permeabilized with 100% methanol. Endogenous EGFR was visualized with an anti-EGFR antibody in combination with anti-rabbit Alexa594. The DNA was stained with Dapi (blue). White box is enlarged in insets. Bar: 10 μm, Insets 1 μm. White arrows indicate colocalization of EGFR and cluster proteins on vesicular structures or the plasma membrane, pink arrows indicate cluster proteins on vesicular structures showing no colocalization with EGFR. The images are representative for three different biological replicates (*n* = 3).

To gain first insight into the potential function of the cluster proteins, we constructed GFP fusion proteins of all 10 large cluster proteins and expressed each of them in human HEp-2 cells to determine their subcellular localization. We used the well characterized EGFR as a marker for the plasma membrane (PM) and for endocytic vesicles (Figure 2). For all proteins harboring no DUF domain (Mbp2, Mbp8) or only the DUF562 domain (Mbp3, Mbp7) we observed an even distribution throughout the cell and the nucleus (Figure 2).

Two proteins carrying only the DUF575 (Mbp1, Mbp6) showed a vesicle-like distribution within the cell and an additional localization to the PM of transfected cells (Figure 2). Interestingly, while expression of Mbp6-GFP led to a complete re-localization of EGFR into ER-like structures and no colocalization was observed, expression of Mbp1-GFP revealed a normal EGFR distribution and we observed colocalization of Mbp1 and EGFR at the PM and on endocytic vesicles (Figure 2). Of the proteins harboring both DUF domains (Mbp4, Mbp9, Mbp11, Mbp13) we were unable to express Mbp13, but we found that while Mbp4 and Mbp11 showed intracellular vesicle-like structures not colocalizing with EGFR, Mbp9 localized to the PM and also in intracellular vesicles both colocalizing with EGFR though on vesicles only partially (Figure 2).

**Figure 3:**
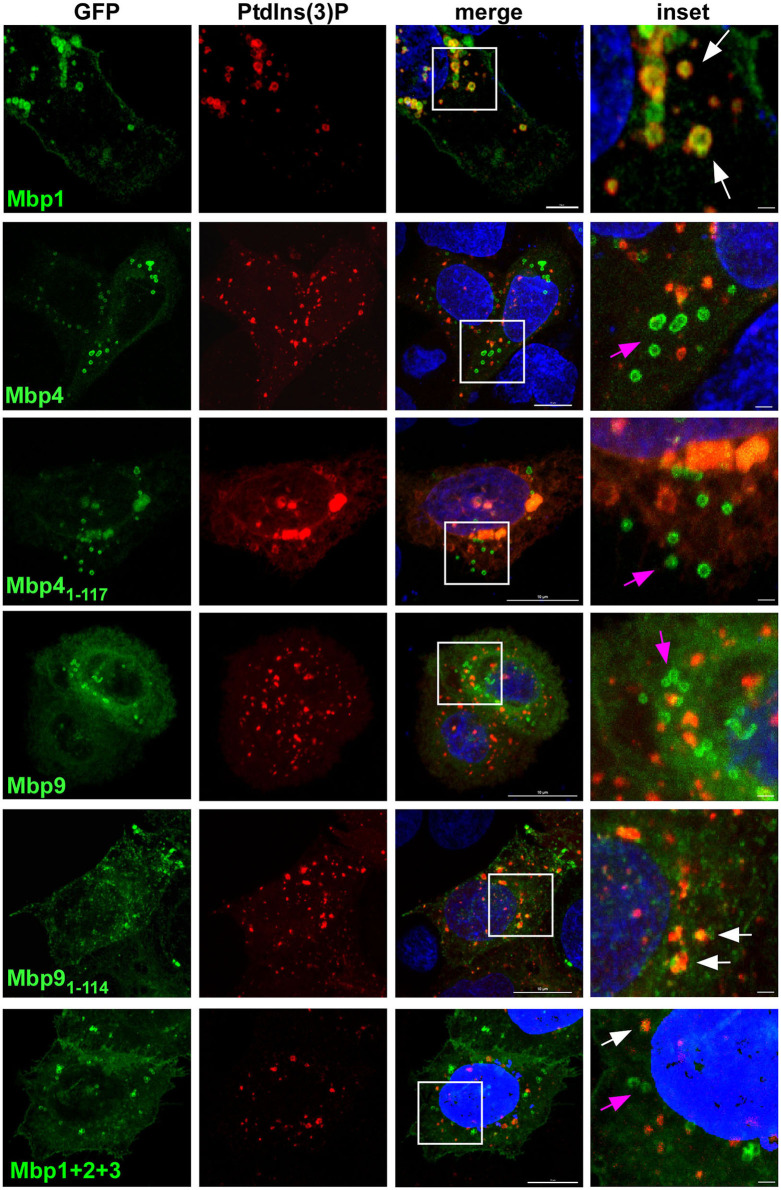
The DUF575-dependent localization to specific membranes is influenced by the presence or absence of the DUF562 domain. Confocal images of cells co-expressing either full length, truncated (only DUF575) or chimera (fusion of three) cluster proteins fused with GFP and 2xFYVE-mCherry, a biosensor for detection of early endosomal membranes positive for PdtIns(3)P. Cells were fixed after 18 h of transfection with 3 % PFA. DNA was visualized with Dapi. White box is enlarged in insets. White arrows indicate colocalization of cluster proteins to vesicular structures positive for PdtIns(3), pink arrows indicate cluster proteins on vesicular structures showing no colocalization. Bar: 10 μm, Insets 1 μm. The images are representative for 3 different biological replicates (*n* = 3).

As the DUF575 domain clearly is important for localization to vesicular structures and to the plasma membrane we next wanted to analyze this phenotype in more depth. Thus, we focused on Mbp1, Mbp4, and Mbp9 as examples of cluster proteins that colocalize with EGFR both on vesicles and at the PM (Mbp1, Mbp9), or that localizes to intracellular vesicles which are not EGFR positive (Mbp4) (Figure 2). We expressed the three full length proteins as well as only the DUF575 domain of Mbp4 and Mbp9 together with mCherry-2xFYVE, a biosensor for phosphoinositide PtdIns(3)P, marker of the early endosome (EE) (Figure 3). For Mbp1 we observed that it colocalized perfectly with EEs indicating a distinct binding of the protein to EE membranes mediated by the DUF575 domain. Interestingly, this colocalization was nearly abolished when we expressed an artificial chimera of Mbp1 fused with Mbp2 and Mbp3 (Mbp1+2+3) that resembles the fusion protein (CPK0979) found in the Koala strain, carrying both DUF domains (Figure 1). Interestingly, this new chimeric protein now containing a DUF562 domain (located in Mbp3), did not change the vesicular phenotype of the chimera but the colocalization to specific EE membranes (Figure 3). *Vice versa* this is also true for Mbp9, harboring both DUF domains, which showed intracellular vesicles which are not EEs; however, if we expressed only the DUF575 domain of Mbp9 (Mbp9_1−114_), the protein now colocalized partially with EE membranes (Figure 3). Moreover, expression of Mbp4 full length harboring both DUF domains, showed the same phenotype as expression of Mbp4_1−117_ (only DUF575) did: vesicles which are not EEs (Figure 3). These findings indicate that DUF575 mediates association with membranes in general, but that specificity for membrane binding is determined by differences in amino acid sequence within the DUF575 and/ or the presence of the DUF562 domain.

### Ectopically Expressed Mbp1 Colocalizes With Rab36 on Early Endosomal Vesicles

**Figure 4:**
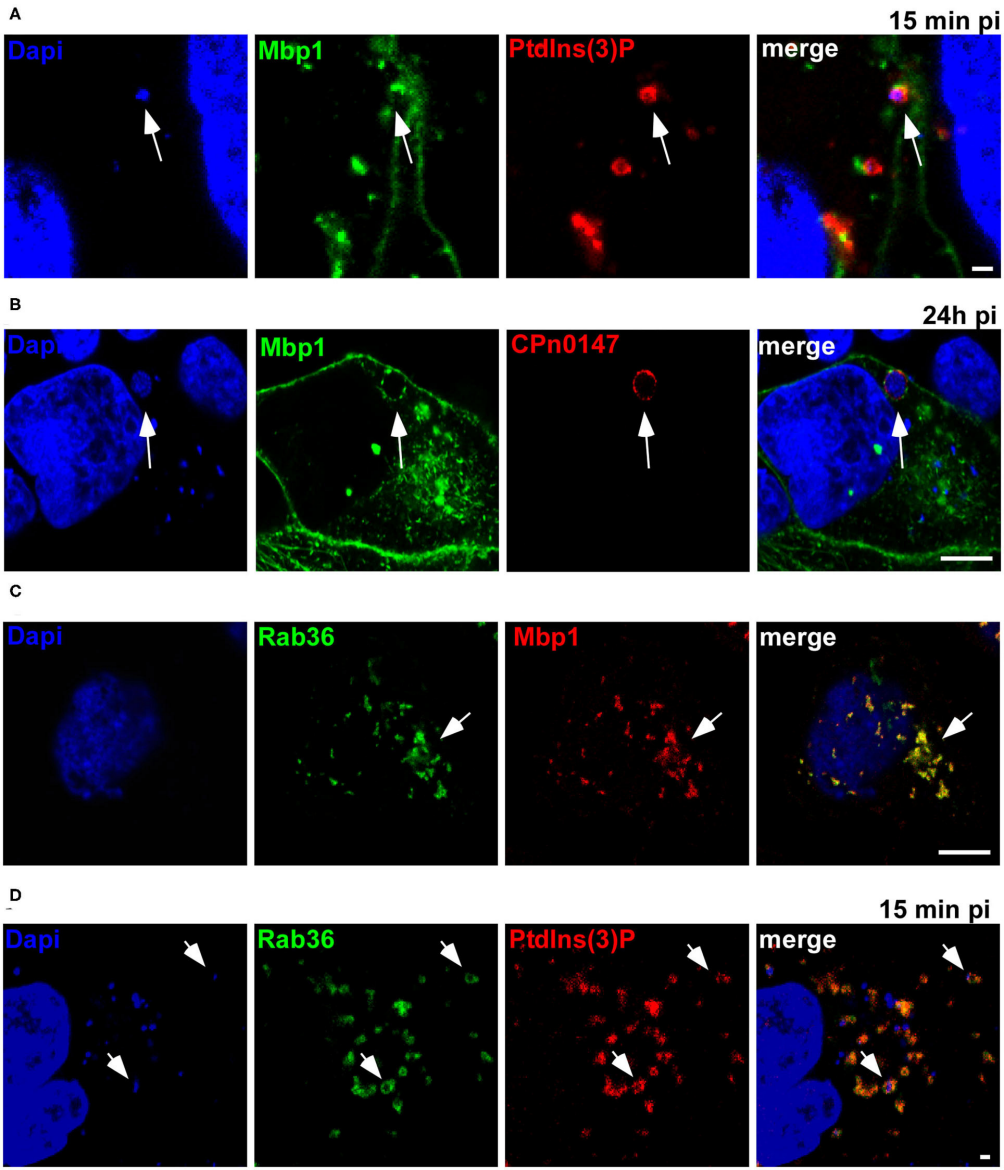
Ectopically expressed Mbp1 colocalizes with Rab36 and both associate with the early inclusion membrane. **(A)** HEp-2 cells expressing Mbp1 and 2xFYVE-mCherry were infected with *Cpn* GiD EBs at an MOI of 5 for 15 min. DNA was visualized with Dapi. White arrows point to Mbp1 associated to EBs enclosed in a PdtIns(3)P-positive membrane **(B)**. Cells expressing Mbp1 were infected with *Cpn* GiD EBs at an MOI of 1 for 24 h, fixed with PFA and permeabilized with methanol. The inclusion membrane was visualized by an anti-CPn0147 antibody in combination with anti-rabbit Alexa594, DNA was visualized with Dapi. White arrows point to Mbp1 colocalizing with CPn0147 on the late inclusion membrane. **(C)** HEp-2 cells were transfected with GFP-Rab36 and Mbp1-mCherry. White arrows indicate colocalization of both proteins in vesicular structures. **(D)** Cells coexpressing GFP-Rab36 and 2xFYVE-mCherry were infected with *Cpn* GiD (MOI 5) for 15 min. DNA was visualized with Dapi. White arrows point to Rab36 associated to the early inclusion positive for PdtIns(3)P. Scale 1 μm. The images are representative for 3 different biological replicates (*n* = 3).

As Mbp1 specifically binds to vesicles that resemble the early *Cpn* inclusion in being EGFR-positive, early endosomes (Molleken and Hegemann, 2017), we next analyzed whether it can colocalize with the chlamydial inclusion during ectopic expression (Figure 4). Indeed, we found Mbp1 nicely colocalizing with the PtdIns(3)P positive inclusions at 15 min pi (Figure 4A) and on the inclusion membrane during later stages of infection (24 h pi, Figure 4B) indicating that the protein can bind to both types of membrane: the early inclusion which is PtdIns(3)P positive and the late one which is not.

Initially, we identified the Mbp cluster based on the limited identity of Mbp1 to the human Rab36, a small GTPase first reported to regulate movement of late endocytic vesicles. Rab36 was shown to be recruited through interaction with Rab35 and MICAL-L1 to recycling endosomes, which implies a broader role for Rab36 in vesicular trafficking (Chen et al., 2010; Kobayashi et al., 2014). Thus, when we coexpressed GFP-Rab36 and Mbp1-mCherry we found a complete colocalization of both proteins on intracellular vesicles (Figure 4C). Moreover, we observed a colocalization of Rab36 with PtdIns(3)P-positive *Cpn* inclusions at 15 min pi (Figure 4D), indicating that this specific Rab protein is also recruited to the early inclusion as we have shown previously for other Rabs like Rab11 or Rab14 (Molleken and Hegemann, 2017).

### A Conserved Sequence in Mbp1 Is Essential for Its Binding to the Early Endosome

**Figure 5:**
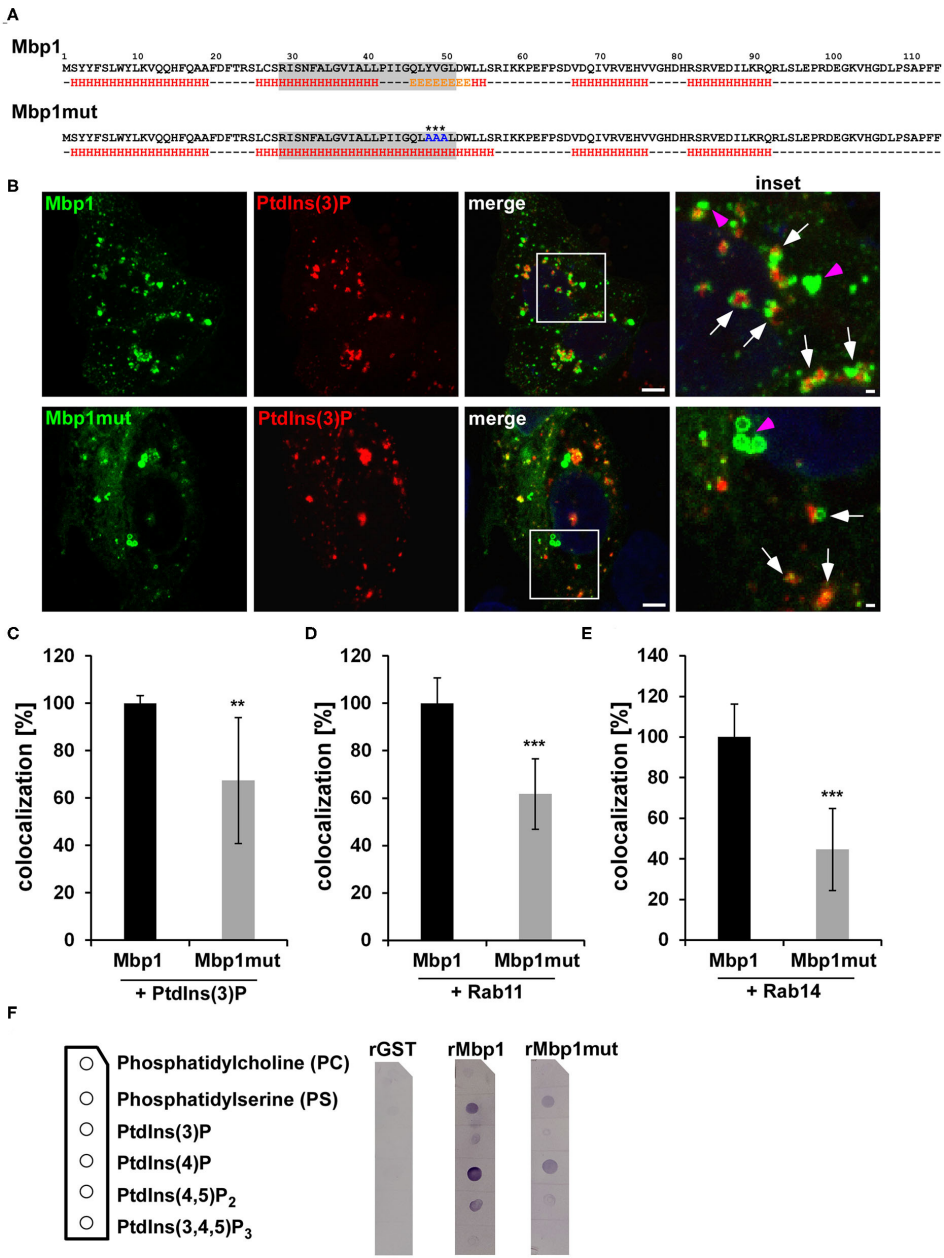
The Mbp1 point mutant (mut) shows reduced association with PtdIns(3)P, Rab11, and Rab14-positive membranes. **(A)** Schematic representation of wild type (Mbp1) and mutant (Mbp1mut) amino acid sequences and their underlying predicted secondary structures using https://npsa-prabi.ibcp.fr/cgi-bin/npsa_automat.pl?page=/NPSA/npsa_gor4.html. H = α-helix E = β-sheet. The gray box displays the transmembrane domain predicted with http://www.cbs.dtu.dk/services/TMHMM/. Asterisks mark the exchanged amino acids in Mbp1mut. **(B)** Representative confocal images of HEp-2 cells coexpressing Mbp1 or Mbp1mut proteins fused to GFP and 2xFYVE-mCherry marking PtdIns(3)P. The white box is enlarged in insets and white arrows indicate colocalization of Mbp1 or Mbp1mut with PdtIns(3)-positive vesicles, pink arrows indicate Mbp1 or Mbp1mut vesicles showing no colocalization. Bar: 5 μm, Insets 1 μm. **(C–E)** Quantification of colocalization of Mbp1 or Mbp1mut with PdtIns(3) **(C)**, with Rab11 **(D)**, and Rab14 **(E)**. Large images of transfected cells were generated and 20 cotransfected cells of 3 different biological replicates were analyzed for colocaliaztion using ImageJ (*n* = 3). ***P ≤ 0.001, **P ≤ 0.01. **(F)** Membrane lipid strip assay in which lipid strips (lipid positions are shown in the scheme) were incubated with 1 μg/ml His-tagged GST, Mbp1 and Mbp1mut protein. After extensive washing of the membrane, protein binding to the indicated lipids was detected by using an anti-His antibody in combination with an alkaline phosphatase coupled anti-mouse antibody. Immunoblot is representative for two biological replicates (*n* = 2).

The colocalization of transiently expressed Mbp1 with EEs and Rab36 prompted us to have a closer look on this protein. By using bioinformatic tools for prediction of transmembrane domains (http://www.cbs.dtu.dk/services/TMHMM/) and general secondary structure predictions (https://npsa-prabi.ibcp.fr/cgi-bin/npsa_automat.pl?page=/NPSA/npsa_gor4.html) we found that all DUF575 domains contain a predicted transmembrane domain (TM) between amino acid 22 and 67, which could be responsible for the observed membrane binding of these cluster proteins. For Mbp1 which colocalizes to the PM and PtdIns(3)P positive endosomes, the predicted TM domain is composed of α-helices and β-strands (Figure 5A). By exchanging amino acids Y_48_V_49_G_50_ (Mbp1) to alanine A_48_A_49_A_50_ (Mbp1mut) we generated a mutant that still contains a predicted TM but loses the β-strands in this region (Figure 5A). To determine whether the β-strand is important for membrane binding specificity of Mbp1, both wild type and mutant Mbp1 were co-expressed with mCherry-2xFYVE, marker for the EE (Figures 5B,C), and either Rab11- or Rab14-mCherry, markers of recycling compartments all found to associate with the early inclusion membrane (Molleken and Hegemann, 2017) (Figures 5D,E). When we quantified the colocalization of Mbp1 with PtdIns(3)P-positive EE vesicles (Figure 5C), with Rab11-positive vesicles (Figure 5D) or with Rab14-positive vesicles (Figure 5E), we observed colocalization of wild type Mbp1 with all three vesicles markers. Interestingly, the Mbp1mut still associated with vesicular structures like the wild type protein did; however, its specificity for particular membrane markers had changed. Quantification revealed a 30 % reduction in colocalization with PtdIns(3)P-positive EEs (Figures 5B,C), a 40 % reduction for vesicles positive for Rab11 (Figure 5D) and a 55 % reduction for those positive for Rab14 (Figure 5E). To further strengthen our findings that the DUF575 is responsible for recognizing and binding to specific phospholipids or phosphoinositides (PtdIns), we tested binding of recombinant proteins to immobilized lipids (Figure 5F). Here, we observed that while the control, GST, did not bind to any of the lipids displayed on the membrane strips, Mbp1 showed significant binding to PS and PtdIns(4)P (Figure 5F). Both lipids locate predominantly to the inner leaflet of the PM (Schink et al., 2016), while PS is also found on RE membranes (Uchida et al., 2011). Moreover, we also observed a weaker binding to PtdIns(3)P and PtdIns(4,5)P_2_ (Figure 5F). While PtdIns(3)P localizes to the membrane of EEs, PtdIns(4,5)P_2_ is found on the inner leaflet of the PM. Interestingly, the Mbp1mut variant which previously showed a significantly reduced binding to EE and RE membranes (Figures 5B,C), is impaired in binding to the lipids bound by the wild type protein (Figure 5F). Together, these findings suggest that the conserved β-strand region within the TM domain is important for membrane binding and plays an important role in directing the association of Mbp1 to vesicles that display an EE or recycling endosome (RE) identity, also found for the early *Cpn* inclusion. Thus, the proteins of the cluster could act as effector proteins binding to specific membranes during early stages of infection.

### Mbp1 and Mbp4 Colocalize With the Outer Membrane Protein Momp on EBs

The Mbp1 transfection/infection experiment found the protein to be localized to the early inclusion 15 min pi (Figures 4A,B) and suggested that Mbp1 might be secreted early in infection. If the cluster proteins are indeed *early* chlamydial effector proteins they should follow certain criteria. (i) They should be expressed at mid-to-late stages of the preceding infection cycle, and stored within the EB in preparation for secretion early in the next infection (like adhesins and early effectors like TarP). (ii) Thus they should be readily accessible by mild detergents treatment of EBs in a protein solubilization assay. (iii) Finally, if secreted via the T3 secretion system, their first 20 amino acids should be recognized for secretion in an heterologous T3 secretion assay, such as the one established in *Shigella flexneri* (Subtil et al., 2001; Hansch et al., 2020).

**Figure 6:**
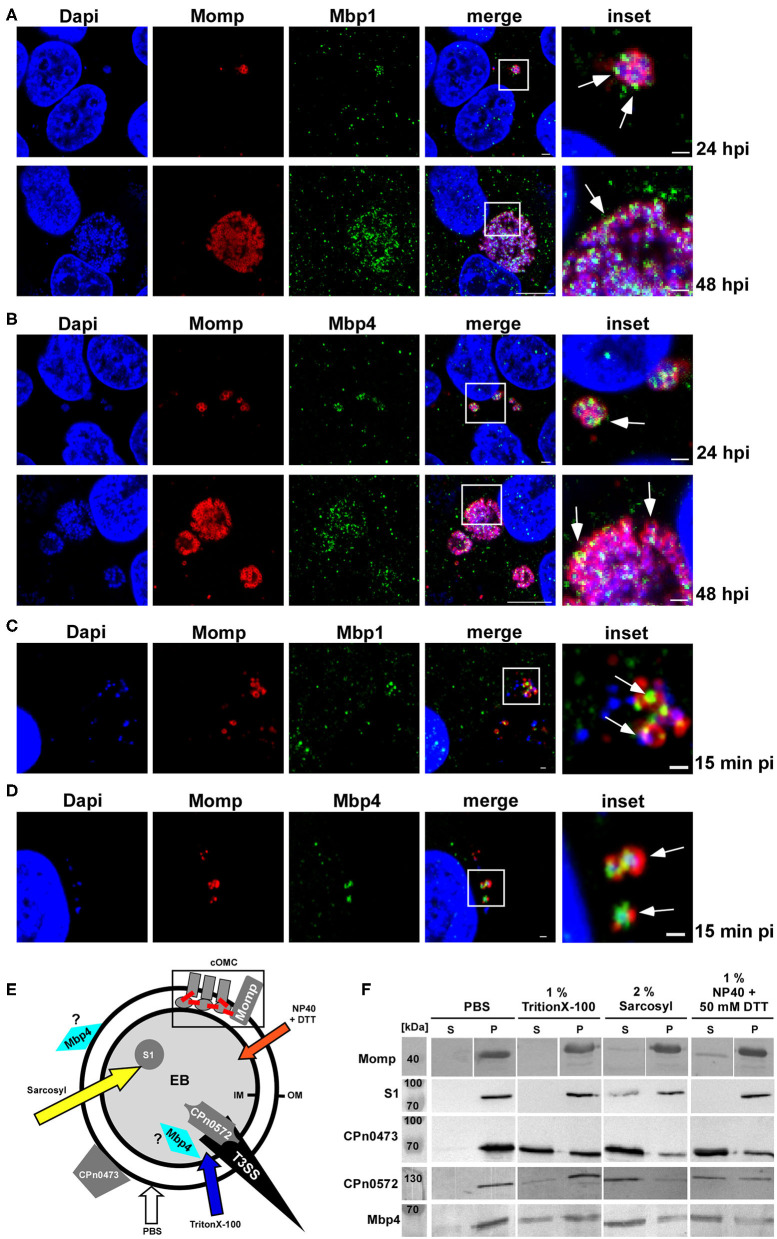
Mbp11 and Mbp4 are expressed mid-/late in the infection cycle, colocalize with Momp and show a solubilization pattern similar to adhesins and type III secreted proteins. **(A–D)** Confocal images of mid-to-late *Cpn* inclusions (24 hpi; 48 hpi) of cells infected with an MOI = 1 **(A,B)** or of adhering *Cpn* EBs at 15 min pi (MOI = 10) **(C,D)** stained with Momp visualized with anti-mouse Alexa594 and specific antibodies for Mbp1 **(A,C)**, Mbp4 **(B,D)**, visualized with anti-rabbit Alexa488. DNA was stained with Dapi. White boxes are shown in insets, white arrows indicate colocalization of Mbp1/Mbp4 with Momp at 15 min, 24 and 48 hpi, respectively. **(A,B)** Bar: 1 μm (15 min pi, 24 hpi and insets). Bar: 10 μm (48 hpi). The images are representative for three different biological replicates (*n* = 3). **(E,F)** Solubilization assay of proteins from purified *Cpn* EBs following exposure to either PBS, 1% Triton X-100, 2% Sarkosyl, or 1% NP40 + 50 mM DTT for 1 h at 37°C. **(E)** Model of the *Cpn* EB indicating the localization of the different tested proteins. The type III secretion needle (T3SS) is shown in black and the outer (OM) and inner (IM) membranes are represented by black circles. The chlamydial outer membrane complex (cOMC) is shown schematically within the gray box. Disulfide bonds are depicted in red. The arrows in different colors indicate the detergents used in the assay. **(F)** Immunoblot analysis of *Cpn* EBs treated with PBS alone or in combination with different detergents. Samples were divided into pellet (*P*) and supernatant (*S*) fractions by high speed centrifugation and analyzed by SDS/PAGE using specific antibodies against the analyzed protein. The immunoblot is representative for two biological replicates (*n* = 2).

First, we studied the localization of cluster proteins during infection. For that we choose Mbp1 harboring the DUF575 domain and Mbp4 carrying both the DUF575 and the DUF562 domains and generated specific antibodies against these proteins. During mid-infection (24 hpi) Mbp1 and Mbp4 colocalized on RBs with the RB-specific, intra-chlamydial DnaK, (Figures S3A,B, left panel) and the outer membrane protein Momp (Figures 6A,B, top panel). Similarly, at 48 hpi both Mbp1 and Mbp4 show colocalization with Momp (Figures 6A,B, bottom panel) and at 72 hpi they are still associated with DnaK-positive RBs (Figures S3A,B, right panel).

We then tested whether the proteins could be detected in association with EBs early in infection. We infected HEp-2 cells, fixed them at 15 min pi and found both Mbps associated with Momp on adhering EBs (Figures 6C,D). In order to support these findings Mbp4 was ectopically expressed in *Ctr*, by generating a plasmid-based 2xMyc tagged DNA construct under the control of the *incD* promotor. We compared Mbp4-2xMyc localization using a Myc antibody by infecting human cells with *Ctr*+Mbp4 or the parental Ctr L2 wild type for 15 min pi and 24 hpi (Figure S3C). Again we used Momp as marker for the outer membrane and indeed we again found a significant colocalization of Momp and Mbp4, especially in attaching EBs 15 min pi (arrow, Figure S3C). Taken together these results show, that both proteins are found on EBs during the adhesion step but are expressed mid-late, which correlates with data from a transcriptome analysis reported by Mäurer et al., showing that *mbp1, mbp2, mbp3*, and *mbp5* from the *Cpn* isolate CWL029 are expressed late and thus were categorized as early proteins (Maurer et al., 2007).

To gain further insight into the subcellular localization of the cluster proteins, we next used a solubilization assay in which purified *Cpn* EBs were treated with either PBS or various mild or harsh detergents and probed for several known chlamydial proteins and for Mbp4 (Figures 6E,F). As member of the chlamydial outer membrane complex carrying disulfide bonds, Momp could only be partially released from the EB by a combination of NP-40 and DTT, whereas the intra-chlamydial protein S1 could only partially be solubilized by strong detergents, such as Sarkosyl (Figures 6E,F). Both the surface localized adhesin CPn0473 and the type III secreted early effector CPn0572 (*Cpn* TarP ortholog), were detectable, albeit in different amounts, in supernatants after mild (Triton X-100) and harsh detergent extraction (Sarcosyl). Mbp4 showed an extraction pattern similar to CPn0473 and CPn0572 and could also be detected in the supernatant after treatment with all tested detergents (Figures 6E,F), indicating that the cluster proteins are either localized on the surface of the EB or in a pre-loaded state in preparation for T3S-mediated secretion. To test for the latter hypothesis we tried to performed a heterologous T3S assay using the first 20 aa of Mbp1 or Mbp4 fused to the Cya (calmodulin-dependent adenylate cyclase) reporter protein to be secreted in *Shigella flexneri* (Subtil et al., 2001). As we failed to express these fusion constructs in *Shigella* (data not shown), we cannot answer whether the cluster proteins could be secreted.

### Proteins of the Cluster Can Bind to Membranes but Are No Adhesins

**Figure 7:**
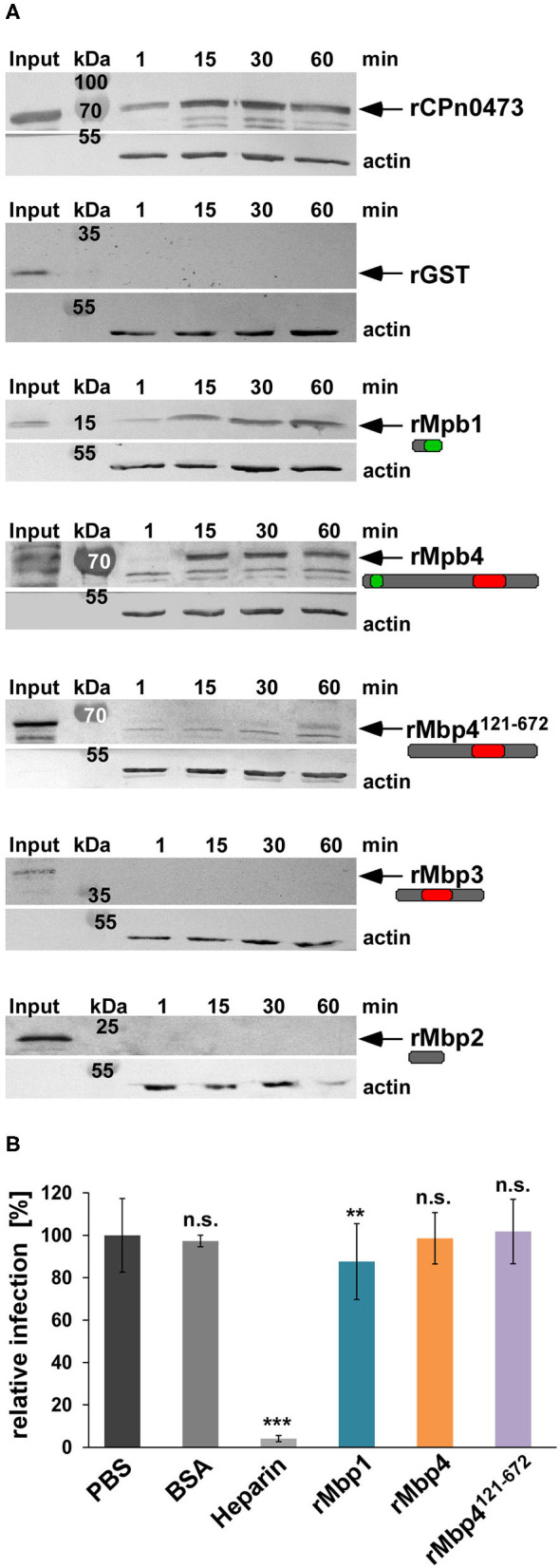
The DUF575 enables cluster proteins to adhere to the plasma membrane. **(A)** Adhesion assay of His-tagged recombinant proteins to HEp-2 cells. HEp-2 cells were incubated with the indicated recombinant proteins at 100 μg/ml for 1 to 60 min. Unbound protein was washed away, cells were lysed and subjected to SDS/PAGE. The amount of bound protein was analyzed on immunoblots with anti-His and secondary anti-mouse AP antibody. Binding capacity was compared to the input level and the actin loading control detected by anti-actin and anti-mouse AP antibody. The known adhesin CPn0473 served as positive, GST as negative control. The arrows indicate the respective protein bands. The immunoblots are representative for three biological replicates (*n* = 3). **(B)** Quantification of infection blocking assays in which HEp-2 cells are incubated with 100 μg/ml of each indicated blocking agent for 60 min. Unbound protein is washed away and cells are incubated with *Cpn* EBs at an MOI = 1 for 2 h, before the medium is replaced. The infection is fixed with methanol after 48 hpi. Relative infectivity was determined by comparing the number of inclusions per human cell and is expressed as a percentage of the number of inclusions determined for the PBS-treated control sample. BSA and heparin were used as negative and positive controls. Data shown are means of 6 biological replicates (*n* = 6). ***P ≤ 0.001, **P ≤ 0.01, n.s. *P* ≤ 0.05.

The cluster proteins Mbp1 and Mbp4 show a specific colocalization to the outer membrane of EBs and RBs and their solubility pattern resembles those of an adhesin or a T3 effector. In order to test whether the proteins have adhesive capacities, we performed adhesion studies in which human HEp-2 cells are tested for binding of selected recombinant (r) cluster proteins (Figure 7A). Interestingly, compared to the controls, the chlamydial adhesin rCPn0473 (Fechtner et al., 2016) and rGST, we observed that only rMbp1 and rMbp4 proteins carrying a DUF575 domain were able to adhere to the human cells, while those carrying only the DUF562 (rMbp3) or no DUF at all (rMbp2) did not bind to the cells (Figure 7A). Furthermore, we could show that the DUF575 domain is essential for adhesion, as rMbp4^121−672^, a DUF575 deletion variant, clearly showed strongly reduced adhesion to HEp-2 (Figure 7A). These data indicate that, as already seen in the ectopic expression of DUF575 containing proteins, this DUF domain plays a critical role in the ability of the proteins to bind to the PM of epithelial cells.

If the cluster proteins are adhesins, pre-incubation of host cells with recombinant proteins would saturate potential receptors and thereby block a subsequent infection as we have previously shown for several chlamydial adhesins like OmcB or Pmp21 (Moelleken and Hegemann, 2008; Moelleken et al., 2010). Thus, we tested whether pre-incubation of HEp-2 cells with rMbp1, rMbp4, or rMbp4^121−672^ had a negative effect on the *Cpn* infection (Figure 7B). Infection levels were normalized to PBS treated cells and compared to cells pre-incubated with BSA, which served as a negative control or cells treated with heparin, a well-described positive control blocking OmcB-mediated adhesion by 96% (Wuppermann et al., 2001) (Figure 7B). In contrast, pre-incubation with rMbp1, rMbp4, or rMbp4^121−672^ (all of which showed some capacity to bind to human cells) had very little (rMbp1) or no influence on the subsequent *Cpn* infection (Figure 7B). These data indicate that although some cluster proteins have the ability to bind to the human PM in a DUF575 dependent manner, they do not play a role in the adhesion process of the EB to the surface of the host cell.

Given our previous results that the DUF575 domain enables cluster proteins to bind to specific membranes during ectopic expression (Figures 2–5), that Mbp1 and Mbp4 are colocalizing with Momp on adhering EBs (Figure 6), but are unable to block a subsequent chlamydial infection as typical chlamydial adhesins do, we conclude that they could be surface localized membrane binding proteins, which use the DUF575 domain to bind to the outer chlamydial membrane and are integral part of the surface of *Cpn* EBs.

## Discussion

Among the *Chlamydiae* with their shared obligate intracellular life cycle, the single species are diverse in terms of host and tissue tropism with some being very restricted to one host such as *Ctr* while others especially those infecting animals like *C. pecorum* have a broad host range. *Cpn* a human pathogen of the upper and lower respiratory tract, that has adapted to a broad variety of tissues as it can be isolated from the respiratory, the cardiovascular system and even the brain, also found a way to infect other warm-blooded animals (Bodetti et al., 2002; Roulis et al., 2013). In the attempt to identify new proteins involved in the early events of the *Cpn* infection we identified the gene cluster *mbp1-mbp13* (*GiD_A_04840-04720*) based on a 27.9% overall identity of one of its predicted products (Mbp1) to the human GTPase Rab36.

The bioinformatic analysis of the gene cluster located between the highly dynamic *pmp* genes *pmp15* and *pmp14*, revealed that it is specific to *Cpn* strains ranging from the koala LPCoLN to human isolates and makes up for 1,12% of the genome coding capacity. As LPCoLN is considered to be the ancestor to the modern human isolates (Myers et al., 2009; Mitchell et al., 2010; Roulis et al., 2013), it is feasible to speculate that initially the zoonotic strain ancestral to the modern koala strain acquired the gene cluster *via* horizontal gene transfer (HGT), which then during evolution and adaptation resulted in the fused or separated genes currently found. Interestingly, comparative genomics of the zoonotic and clinical *Cpn* strains revealed that one member of the cluster (*mbp13*) is among a small group of genes showing a positive selective pressure to be maintained during transition from animal to human isolates (Weinmaier et al., 2015). Furthermore, the distribution of SNPs and IN/ DELs within the cluster genes correlates with tissue specificity of clinical strains isolated from the respiratory tract or the cardiovascular system (Weinmaier et al., 2015), indicating an ongoing adaptation to specific niches and tissues.

We used ectopic expressed cluster proteins fused with GFP to analyze the potential function and subcellular localization of the proteins and found that the conserved DUF575 domains play an important role in the association of the proteins to the host endomembrane system. The analysis of proteins carrying both DUFs and different deletion variants revealed that while the DUF575 domains, all harboring a predicted TM domain, enable the proteins to bind to membranes in general, specificity of membrane binding is modulated by presence or absence of the DUF562 domain. Mbp1, founder of the cluster, carrying only the DUF575, showed a very distinct colocalization with EGFR at the PM and on intracellular vesicles, which are EEs with a specific PtdIns(3)P membrane identity. Furthermore, we found Mbp1 to associate with different Rab GTPases like Rab11 or Rab14, both specifically recruited to the early *Cpn* inclusion (Molleken and Hegemann, 2017) and, very interestingly, with Rab36, which shares a 27.9% overall identity with Mbp1. Rab36 is one of the less-known Rab family members; however, available data indicate a possible regulatory role in the spatial distribution of late endosomes and lysosomes and that it localizes to recycling endosomes upon recruitment via Rab35 and MICAL-L1 (Chen et al., 2010). We could show not only colocalization of Rab36 and Mbp1, but also found both recruited to early inclusion during ectopic expression, indicating that both share a mechanism to bind to this specific membrane within the cell.

By using secondary structure predictions and subsequent mutational analysis we were able to identify an intrinsic β-strand sequence within the predicted TM of Mbp1 to be important for the specific binding to EE membranes and association with Rab11 and Rab14 during ectopic expression, which was clearly reduced in the mutant. These findings are supported by the analysis of both recombinant wild type and mutant protein for their ability to bind to phospholipids immobilized on membranes. The mutant was less able to bind to those lipids compared to the wild type protein, thus indicating that the DUF575 contains intrinsic sequences needed for binding to specific membranes. As the cluster contains at least 6 proteins carrying a DUF575 domain, this suggests that all of them can specifically interact with membranes of various types and that the presence of the second DUF domain may influence this binding.

Concerning the function of the cluster proteins during the *Cpn* infection we could show that both Mbp1 and Mbp4 are expressed mid to late during the infection and can colocalize with Momp on RBs and on EBs within the first 15 min of infection. In addition, Mbp4 subcellular localization was analyzed in a biochemical solubilization assay of purified EBs, in which it was found associated either with the EB surface like an adhesin or with the T3SS as a preloaded T3S substrate ready for secretion. These results suggest either a potential T3 secretion, which we could not verify thus far, or that the cluster proteins are important part of the chlamydial surface and/or the cOMC (chlamydial outer membrane complex). Other proteins localizing to the cOMC like Momp or the adhesins OmcB, LipP, or the Pmps are highly immunogenic (Su et al., 1990; Moelleken and Hegemann, 2008; Moelleken et al., 2010; Galle et al., 2019). However, none of the Mbp cluster proteins was found in a genome-wide approach identifying immunogenic *Cpn* surface proteins (Montigiani et al., 2002). Despite the fact that recombinant cluster proteins have the ability to adhere to human epithelial cells in a DUF575 dependent manner, they are unable to interfere with a subsequent infection when added to human cells prior to infection. Although the DUF575 can mediate Mbp binding to phospholipids for example of the PM or the RE, the proteins are not involved in the adhesion process of the bacteria to the cell.

Therefore, we hypothesize that the cluster proteins are either on the outer membrane of EBs and interact with host membranes during adhesion and internalization or they are secreted during these early events and can interact with the nascent inclusion membrane.

## Materials and Methods

### Antibodies

Primary antibodies against DnaK were kindly provided by S. Birkelund (Birkelund et al., 1990). Antibodies directed against recombinant full length Mbp4 and Mbp1 were produced by Eurogentec (Seraing, Belgium) and purified for this study. Anti-CPn0572, -CPn0473, -CPn0147, and -Momp antibodies were generated in our lab. The anti-GST (1:1000) antibody was purchased from Cell Signaling Technology (Frankfurt am Main, Germany), the anti-His antibody (1:2500) from Qiagen (Hilden, Germany), the anti-Myc from Chromotek (Munich, Germany), the anti-EGFR antibody (1:400) from Thermo-Fisher Scientific (Waltham, Massachusetts, USA), and the anti-β-actin antibody (1:2500) from Merck Millipore (Darmstadt, Germany). Secondary anti-rat/rabbit/mouse antibodies coupled to Alexa 488 and 594 for immunofluorescence (1:200) were purchased from Thermo-Fisher Scientific. Secondary anti-rabbit/mouse antibodies coupled to AP conjugate for immunoblot analysis were obtained from Promega (1:7500) (Fitchburg, Wisconsin, USA).

### Cloning Procedures

The genes *mbp1* to *mbp13* were amplified from *Cpn* GiD DNA by PCR and integrated into pKM55 (C-terminal GFP) or pSL4 (C-terminal 10x His-Tag). Coding sequences for Rab11-a and Rab14 were amplified from pEGFP-C2-Rab11a, and pEGFP-C1-Rab14, respectively, and for 2xFYVE from pGFP-FYVE(2x), and each cDNA was integrated into pAE66 (N-terminal mCherry). *mbp4* was amplified *Cpn* GiD DNA by PCR and integrated into pKM213 (IncDPromoter-2xMyc Tag-IncDTerminator) which was generated based on p2TK2–SW2 IncDPromoter-RSGFP-IncDTerminator, kindly provided by Isabelle Derre to generate pKM266. All constructs were generated by homologous recombination in *S. cerevisiae* and verified by sequencing.

### Growth of *Chlamydia* and Human Cell Lines

HEp-2 cells were cultured in DMEM medium supplemented with 10% fetal calf serum (FCS), MEM vitamins and non-essential amino acids (Thermo Fisher Scientific). *Cpn* GiD EBs were propagated in HEp-2 cells (ATCC: CCL-23). Elementary bodies (EBs) were purified using a 30% gastrografin solution (Bayer; Leverkusen, Germany) and stored in SPG buffer (220 mM sucrose, 3.8 mM KH_2_PO_4_, 10.8 mM Na_2_HPO_4_, 4.9 mM L-glutamine).

### Transformation of *Ctr* L2

Transformation of Ctr L2 was performed according protocols from (Mueller et al., 2016). Briefly, EBs were incubated with 100 μl CaCl_2_ buffer and 5 μg of pKM266 non-methylated plasmid DNA for 20 min at room temperature. The mixture was added into 5 ml DMEM supplemented with 1.2 μg/ml cycloheximid and centrifuged onto a 25 cm^2^ confluent monolayers of HEp-2 cells at 2,800 rpm and 37°C. After 12 hpi media was exchanged to media containing 1.2 μg/ml cycloheximid and 1 μg/ml Penicillin. Cells were passaged 3 times under selective pressure until positive transformed *Ctr* EBs were harvested and purified.

### Infection Experiments

HEp-2 cells were exposed to *Cpn* GiD EBs (MOI 1) by centrifugation at 2800 rpm (Rotanda, Hettich) for 15 min (early infection time points) or 60 min (mid-/ late infection time points 24 h to 72 h) at 37°C. After centrifugation, cells were shifted to 37°C to initiate infection, and grown under 6% CO_2_ for periods ranging from 0 min to 72 h prior to fixation with 3% paraformaldehyde in PBS (PFA) for 10 min at RT.

### Transfection Experiments

HEp-2 cells were grown in 24-well plates (Sarstedt; Nümbrecht, Germany) on coverslips for 24 h in complete medium with FCS. For transfection, fresh medium without FCS was added and cells were transfected for 18 h using TurboFect (Thermo-Fisher Scientific). The cells were then fixed with 3% paraformaldehyde and analyzed by confocal microscopy (Nikon Confocal C2plus).

### Solubilization Assay

*Cpn* EBs (1 × 10^7^) were centrifuged for 30 min at 4°C and 15,000 × g. The pellet was resuspended in PBS (sonification bath) and incubated with PBS (control), 1 % Triton-X 100, 2% Sarkosyl, or 1% NP-40 + 50 mM DTT at 37°C for 1 h with a short burst of sonification every 10 min, then centrifuged for 1 h at 4°C and 100,000 × g. The supernatant was recovered and the pellet resuspended in PBS. Samples of pellet and supernatant fractions were subjected to SDS/PAGE and analyzed with specific antibodies after Western blotting.

### Adhesion Assay

Confluent monolayers of HEp-2 cells were grown in 24-well plates (Sarstedt). The spent medium was removed and replaced with fresh DMEM (250 μl) containing recombinant His-tagged protein (100 μg/ml) and incubated at 37°C in the presence of 6% CO_2_. The medium was removed after 1, 15, 30, and 60 min and the unbound protein washed away with HBSS. The cells were then detached with cell dissociation solution (Merck Millipore). The suspension was transferred to a new reaction tube and centrifuged for 5 min at 1,000 × g. Supernatant was discarded and the pellet resuspended in PBS. The sample was analyzed by SDS/PAGE and the proteins detected with a specific anti-His antibody.

### Infection Blocking Assay

HEp-2 cells were grown as described in the previous paragraph. DMEM medium was removed and 250 μl (100 μg/ml) of recombinant protein in DMEM medium was added to the cells and incubated at 37°C and 6% CO_2_ for 1 h. Unbound protein was washed away, the cells infected with *Cpn* GiD (MOI 1) and incubated for 2 h. EB solution was removed, replaced with fresh medium supplemented with cycloheximide (1.2 μg/ml) and incubated for 48 h. Cells were fixed with 3% PFA, permeabilized with 100% methanol and the inclusion stained with anti-CPn0147 antibody. DNA was visualized with DAPI. The number of nuclei and inclusions was quantified by confocal imaging.

### Protein Purification

His-tagged proteins were expressed in *E. coli* BL21 and purified under denaturing conditions (8 M urea, 0.1 M NaH_2_PO_4_, 10 mM Tris/HCl) from cell lysates by affinity chromatography on Ni-NT agarose columns (Merck Millipore) and performed as recommended by the manufacturer. Proteins were eluted with 500 mM imidazole (Acros Organics), dialyzed in PBS (137 mM NaCl, 2.7 mM KCl, 10 mM Na_2_HPO_4_, 1.8 mM KH_2_PO_4_, pH7.4) at 4°C and protein concentrations were determined using the Bradford assay (Bio Rad).

### Lipid Strip Assay

Aliquots (1.5 μg) of test lipids were spotted on a PDVF membrane (Merck Millipore) and left to dry at RT for 1 h. The membrane was then exposed to a blocking solution [3% BSA (fatty acid free, Serva; Heidelberg, Germany) + 0.1% Tween (w/v)] and incubated overnight at 4°C with 2 μg/ml of the test protein. After washing with PBS-T (1x PBS pH 7.4 + 0.1% Tween), binding of the protein was analyzed with the appropriate anti-His antibody and visualized by anti-mouse antibody coupled to alkaline phosphatase.

### Immunofluorescence Staining

Transfected and infected HEp-2 cells were fixed at the indicated time points with 3% paraformaldehyde in PBS (PFA) for 10 min, then washed three times with HBSS and permeabilized with either 100% methanol for 10 min at room temperature or with 2% saponin (Merck) in PBS for 20 min at 30°C. Cells were analyzed for the subcellular localization of proteins from the 04840-04720 cluster by confocal microscopy (Nikon Confocal C2plus). Primary antibodies were diluted in PBS or 0.5% saponin solution, applied to the cells, and incubated at 30°C for 30 min. Cells were washed three times with PBS with or without 0.5% saponin, and then incubated with secondary antibody anti-rabbit/mouse Alexa488/Alexa594 at 30°C for 30 min. Dapi was used to visualize DNA.

### Quantification of Immunofluorescence Images

Confocal images (4 × 4 stitched multiple adjacent frames) were opened in ImageJ. Quantification of Mbp1 or Mbp1mut colocalization with vesicle markers was performed first by marking the GFP positive Mbp1 or Mbp1mut vesicles in the green channel as regions of interest (ROIs). After that colocalization of red signals (Rab11, Rab14, 2xFYVE fused to mCherry) with green ROIs was counted in 20 cotransfected cells in three biological replicates.

### Microscopy and Image Processing

Immunofluorescence microscopy was performed on an inverse Nikon TiE Live Cell Confocal C2plus equipped with a 100x TIRF objective and a C2 SH C2 Scanner. All images and image-related measurements were generated with Nikon Element software.

### Bioinformatic Analysis

Sequence comparisons of proteins of the *Cpn* GiD cluster (Mbp1-Mbp13) were carried out with MUSCLE: https://www.ebi.ac.uk/Tools/msa/muscle/. The identity between the proteins was determined by using the Clustal W output of the MUSCLE alignment tool. Secondary-structure predictions were carried out with Jpred 4: http://www.compbio.dundee.ac.uk/jpred/.

### Statistical Analysis

The data represent the means (±*SD*) of *n* experiments. A Student's *t*-test was chosen for simple paired analysis between two groups. ^***^P ≤ 0.001, ^**^P ≤ 0.01. A *P* ≤ 0.05 was considered non-significant (n.s.).

### Accession Numbers

*Homo sapiens* Epidermal growth factor receptor (EGFR): NM_005228.3
*Homo sapiens* Rab11a: AF000231.1
*Homo sapiens* Rab14: NM_016322.3
*Homo sapiens* Rab36: Isoform CRA_b Accession: EAW59562.1
*Chlamydia pneumoniae* GiD: LN847009.1
*Chlamydia pneumoniae* J138: NC_002491.1
*Chlamydia pneumoniae* AR39: NC_002180.1
*Chlamydia pneumoniae* CWL029: NC_000922.1
*Chlamydia pneumoniae* CV14: NZ_LN846996.1
*Chlamydia pneumoniae* Wien1: NZ_LN846980.1
*Chlamydia pneumoniae* LPCoLN: NC_017285.1

## Data Availability Statement

All datasets generated for this study are included in the article/Supplementary Material.

## Author Contributions

CB, KM, and JH designed the experiments and wrote the manuscript. CB and KM performed, evaluated, quantified the experiments, collected the data, and prepared all figures. All authors contributed to the article and approved the submitted version.

## Conflict of Interest

The authors declare that the research was conducted in the absence of any commercial or financial relationships that could be construed as a potential conflict of interest.

## References

[B1] Birkelund S. Lundemose A. G. Christiansen G. (1990). The 75-kilodalton cytoplasmic *Chlamydia trachomatis* L2 polypeptide is a DnaK-like protein. Infect. Immun. 58, 2098–2104. 10.1128/IAI.58.7.2098-2104.1990 2365454 PMC258782

[B2] Bodetti T. J. Jacobson E. Wan C. Hafner L. Pospischil A. Rose K.. (2002). Molecular evidence to support the expansion of the hostrange of *Chlamydophila pneumoniae* to include reptiles as well as humans, horses, koalas and amphibians. Syst. Appl. Microbiol. 25, 146–152. 10.1078/0723-2020-00086 12086181

[B3] Braun C. U. (2019). Characterization of Effector Proteins Involved in the Early Chlamydia pneumoniae Infection. Düsseldorf: Heinrich Heine University.

[B4] Campa C. C. Margaria J. P. Derle A. Del Giudice M. De Santis M. C. Gozzelino L.. (2018). Rab11 activity and PtdIns(3)P turnover removes recycling cargo from endosomes. Nat. Chem. Biol. 14, 801–810. 10.1038/s41589-018-0086-4 29915378

[B5] Chen L. Hu J. Yun Y. Wang T. (2010). Rab36 regulates the spatial distribution of late endosomes and lysosomes through a similar mechanism to Rab34. Mol. Membr. Biol. 27, 23–30. 10.3109/09687680903417470 19961360

[B6] Chi E. Y. Kuo C. C. Grayston J. T. (1987). Unique ultrastructure in the elementary body of *Chlamydia* sp. strain TWAR. J. Bacteriol. 169, 3757–3763. 10.1128/JB.169.8.3757-3763.1987 3611029 PMC212462

[B7] Clifton D. R. Fields K. A. Grieshaber S. S. Dooley C. A. Fischer E. R. Mead D. J.. (2004). A chlamydial type III translocated protein is tyrosine-phosphorylated at the site of entry and associated with recruitment of actin. Proc. Natl. Acad. Sci. U. S. A. 101, 10166–10171. 10.1073/pnas.0402829101 15199184 PMC454183

[B8] Fechtner T. Galle J. N. Hegemann J. H. (2016). The novel chlamydial adhesin CPn0473 mediates the lipid raft-dependent uptake of *Chlamydia pneumoniae*. Cell. Microbiol. 18, 1094–1105. 10.1111/cmi.12569 26780295 PMC5067637

[B9] Galle J. N. Fechtner T. Eierhoff T. Romer W. Hegemann J. H. (2019). A *Chlamydia pneumoniae* adhesin induces phosphatidylserine exposure on host cells. Nat. Commun. 10:4644. 10.1038/s41467-019-12419-8 31604911 PMC6789132

[B10] Hahn D. L. Dodge R. W. Golubjatnikov R. (1991). Association of *Chlamydia pneumoniae* (strain TWAR) infection with wheezing, asthmatic bronchitis, and adult-onset asthma [see comments]. JAMA 266, 225–230. 10.1001/jama.1991.03470020051031 2056624

[B11] Hansch S. Spona D. Murra G. Kohrer K. Subtil A. Furtado A. R.. (2020). Chlamydia-induced curvature of the host-cell plasma membrane is required for infection. Proc. Natl. Acad. Sci. U. S. A. 117, 2634–2644. 10.1073/pnas.1911528117 31964834 PMC7007526

[B12] Jantos C. A. Heck S. Roggendorf R. Sen-Gupta M. Hegemann J. H. (1997). Antigenic and molecular analyses of different *Chlamydia pneumoniae* strains. J. Clin. Microbiol. 35, 620–623. 10.1128/JCM.35.3.620-623.1997 9041400 PMC229638

[B13] Jewett T. J. Miller N. J. Dooley C. A. Hackstadt T. (2010). The conserved Tarp actin binding domain is important for chlamydial invasion. PLoS Pathog. 6:e1000997. 10.1371/journal.ppat.1000997 20657821 PMC2904776

[B14] Junutula J. R. De Maziere A. M. Peden A. A. Ervin K. E. Advani R. J. Van Dijk S. M.. (2004). Rab14 is involved in membrane trafficking between the Golgi complex and endosomes. Mol. Biol. Cell 15, 2218–2229. 10.1091/mbc.e03-10-0777 15004230 PMC404017

[B15] Kobayashi H. Etoh K. Ohbayashi N. Fukuda M. (2014). Rab35 promotes the recruitment of Rab8, Rab13 and Rab36 to recycling endosomes through MICAL-L1 during neurite outgrowth. Biol. Open 3, 803–814. 10.1242/bio.20148771 25086062 PMC4163657

[B16] Lindsay A. J. Hendrick A. G. Cantalupo G. Senic-Matuglia F. Goud B. Bucci C.. (2002). Rab coupling protein (RCP), a novel Rab4 and Rab11 effector protein. J. Biol. Chem. 277, 12190–12199. 10.1074/jbc.M108665200 11786538

[B17] Madshus I. H. Stang E. (2009). Internalization and intracellular sorting of the EGF receptor: a model for understanding the mechanisms of receptor trafficking. J. Cell Sci. 122, 3433–3439. 10.1242/jcs.050260 19759283

[B18] Maurer A. P. Mehlitz A. Mollenkopf H. J. Meyer T. F. (2007). Gene expression profiles of *Chlamydophila pneumoniae* during the developmental cycle and iron depletion-mediated persistence. PLoS Pathog. 3:e83. 10.1371/journal.ppat.0030083 17590080 PMC1894823

[B19] Mitchell C. M. Hovis K. M. Bavoil P. M. Myers G. S. Carrasco J. A. Timms P. (2010). Comparison of koala LPCoLN and human strains of *Chlamydia pneumoniae* highlights extended genetic diversity in the species. BMC Genomics 11:442. 10.1186/1471-2164-11-442 20646324 PMC3091639

[B20] Miyashita N. Kanamoto Y. Matsumoto A. (1993). The morphology of *Chlamydia pneumoniae*. J. Med. Microbiol. 38, 418–425. 10.1099/00222615-38-6-418 8510134

[B21] Moelleken K. Hegemann J. H. (2008). The *Chlamydia* outer membrane protein OmcB is required for adhesion and exhibits biovar-specific differences in glycosaminoglycan binding. Mol. Microbiol. 67, 403–419. 10.1111/j.1365-2958.2007.06050.x 18086188 PMC2229832

[B22] Moelleken K. Schmidt E. Hegemann J. H. (2010). Members of the Pmp protein family of *Chlamydia pneumoniae* mediate adhesion to human cells via short repetitive peptide motifs. Mol. Microbiol. 78, 1004–1017. 10.1111/j.1365-2958.2010.07386.x 21062373 PMC2997323

[B23] Mölleken K. Becker E. Hegemann J. H. (2013). The *Chlamydia pneumoniae* invasin protein Pmp21 recruits the EGF receptor for host cell entry. PLoS Pathog. 9:e1003325. 10.1371/journal.ppat.1003325 23633955 PMC3635982

[B24] Molleken K. Hegemann J. H. (2017). Acquisition of Rab11 and Rab11-Fip2-a novel strategy for *Chlamydia pneumoniae* early survival. PLoS Pathog. 13:e1006556. 10.1371/journal.ppat.1006556 28787457 PMC5560749

[B25] Montigiani S. Falugi F. Scarselli M. Finco O. Petracca R. Galli G.. (2002). Genomic approach for analysis of surface proteins in *Chlamydia pneumoniae*. Infect. Immun. 70, 368–379. 10.1128/IAI.70.1.368-379.2002 11748203 PMC127649

[B26] Mueller K. E. Wolf K. Fields K. A. (2016). Gene Deletion by fluorescence-reported allelic exchange mutagenesis in chlamydia trachomatis. mBio 7, e01817–01815. 26787828 10.1128/mBio.01817-15 PMC4725004

[B27] Myers G. S. Mathews S. A. Eppinger M. Mitchell C. O'brien K. K. White O. R.. (2009). Evidence that human *Chlamydia pneumoniae* was zoonotically acquired. J. Bacteriol. 191, 7225–7233. 10.1128/JB.00746-09 19749045 PMC2786552

[B28] Roulis E. Polkinghorne A. Timms P. (2013). *Chlamydia pneumoniae*: modern insights into an ancient pathogen. Trends Microbiol. 21, 120–128. 10.1016/j.tim.2012.10.009 23218799

[B29] Schink K. O. Tan K. W. Stenmark H. (2016). Phosphoinositides in control of membrane dynamics. Annu. Rev. Cell Dev. Biol. 32, 143–171. 10.1146/annurev-cellbio-111315-125349 27576122

[B30] Spano S. Galan J. E. (2018). Taking control: hijacking of Rab GTPases by intracellular bacterial pathogens. Small GTPases 9, 182–191. 10.1080/21541248.2017.1336192 28632996 PMC5902217

[B31] Stein M. P. Muller M. P. Wandinger-Ness A. (2012). Bacterial pathogens commandeer Rab GTPases to establish intracellular niches. Traffic 13, 1565–1588. 10.1111/tra.12000 22901006 PMC3530015

[B32] Stroupe C. (2018). This is the end: regulation of rab7 nucleotide binding in endolysosomal trafficking and autophagy. Front. Cell Dev. Biol. 6:129. 10.3389/fcell.2018.00129 30333976 PMC6176412

[B33] Su H. Watkins N. G. Zhang Y. X. Caldwell H. D. (1990). *Chlamydia trachomatis*-host cell interactions: role of the chlamydial major outer membrane protein as an adhesin. Infect. Immun. 58, 1017–1025. 10.1128/IAI.58.4.1017-1025.1990 2318528 PMC258576

[B34] Subtil A. Parsot C. Dautry-Varsat A. (2001). Secretion of predicted Inc proteins of *Chlamydia pneumoniae* by a heterologous type III machinery. Mol. Microbiol. 39, 792–800. 10.1046/j.1365-2958.2001.02272.x 11169118

[B35] Uchida Y. Hasegawa J. Chinnapen D. Inoue T. Okazaki S. Kato R.. (2011). Intracellular phosphatidylserine is essential for retrograde membrane traffic through endosomes. Proc. Natl. Acad. Sci. U. S. A. 108, 15846–15851. 10.1073/pnas.1109101108 21911378 PMC3179068

[B36] Weigele B. A. Orchard R. C. Jimenez A. Cox G. W. Alto N. M. (2017). A systematic exploration of the interactions between bacterial effector proteins and host cell membranes. Nat. Commun. 8:532. 10.1038/s41467-017-00700-7 28912547 PMC5599653

[B37] Weinmaier T. Hoser J. Eck S. Kaufhold I. Shima K. Strom T. M.. (2015). Genomic factors related to tissue tropism in *Chlamydia pneumoniae* infection. BMC Genomics 16:268. 10.1186/s12864-015-1377-8 25887605 PMC4489044

[B38] Wolf K. Fischer E. Hackstadt T. (2000). Ultrastructural analysis of developmental events in *Chlamydia pneumoniae*-infected cells. Infect. Immun. 68, 2379–2385. 10.1128/IAI.68.4.2379-2385.2000 10722649 PMC97433

[B39] Wuppermann F. N. Hegemann J. H. Jantos C. A. (2001). Heparan sulfate-like glycosaminoglycan is a cellular receptor for *Chlamydia pneumoniae*. J. Infect. Dis. 184, 181–187. 10.1086/322009 11424015

[B40] Zrieq R. Braun C. Hegemann J. H. (2017). The *Chlamydia pneumoniae* tarp ortholog CPn0572 stabilizes host F-actin by displacement of cofilin. Front. Cell. Infect. Microbiol. 7:511. 10.3389/fcimb.2017.00511 29376031 PMC5770662

