# Supplementary figures and images for: Insights Into a Chlamydia pneumoniae-Specific Gene Cluster of Membrane Binding Proteins
Source: Front Cell Infect Microbiol. 2020 Oct 21;10:565808. doi: 10.3389/fcimb.2020.565808 (PMC7609445; doi:10.3389/fcimb.2020.565808)

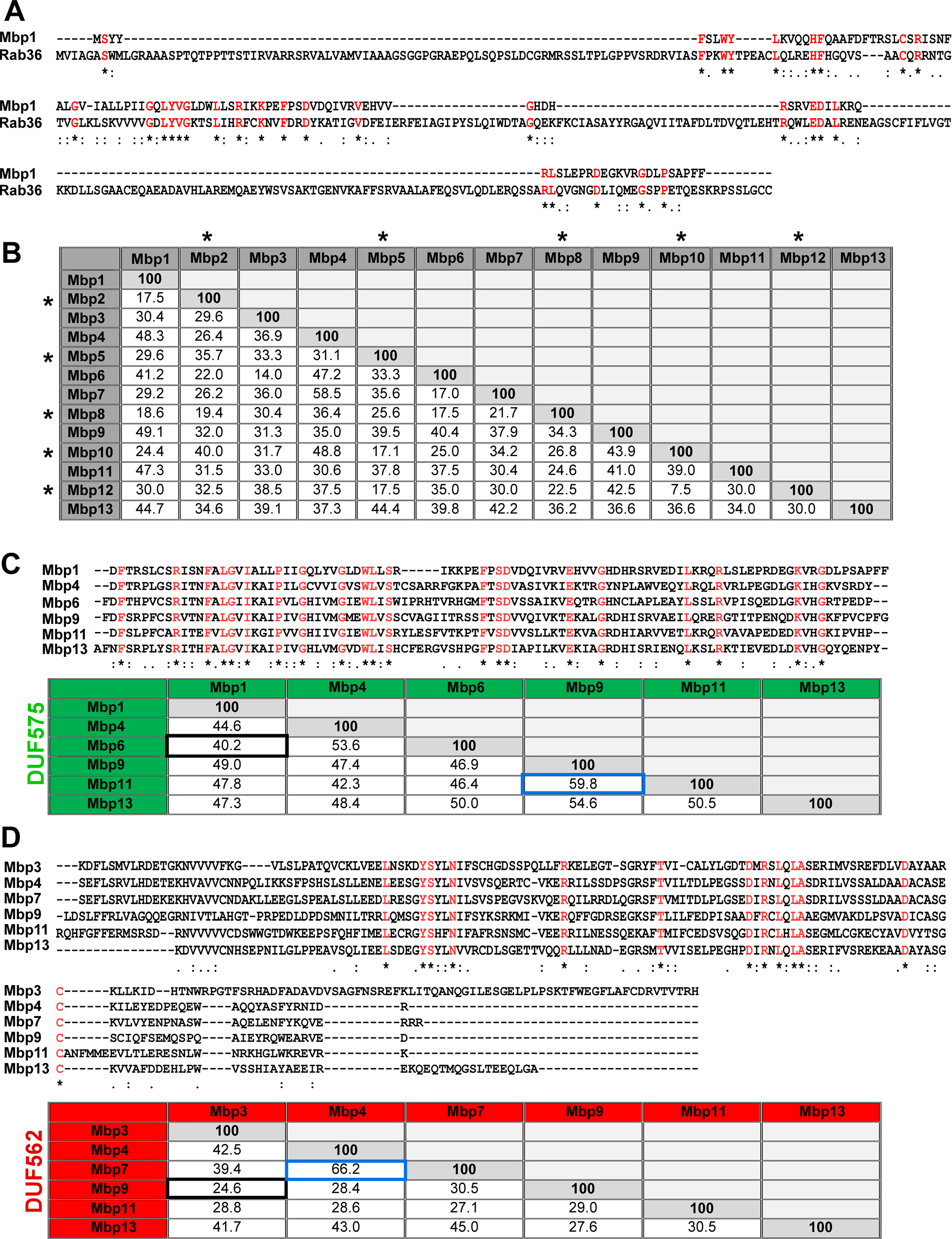

Supplement: Supplementary file 1 [file Image_1.TIF]

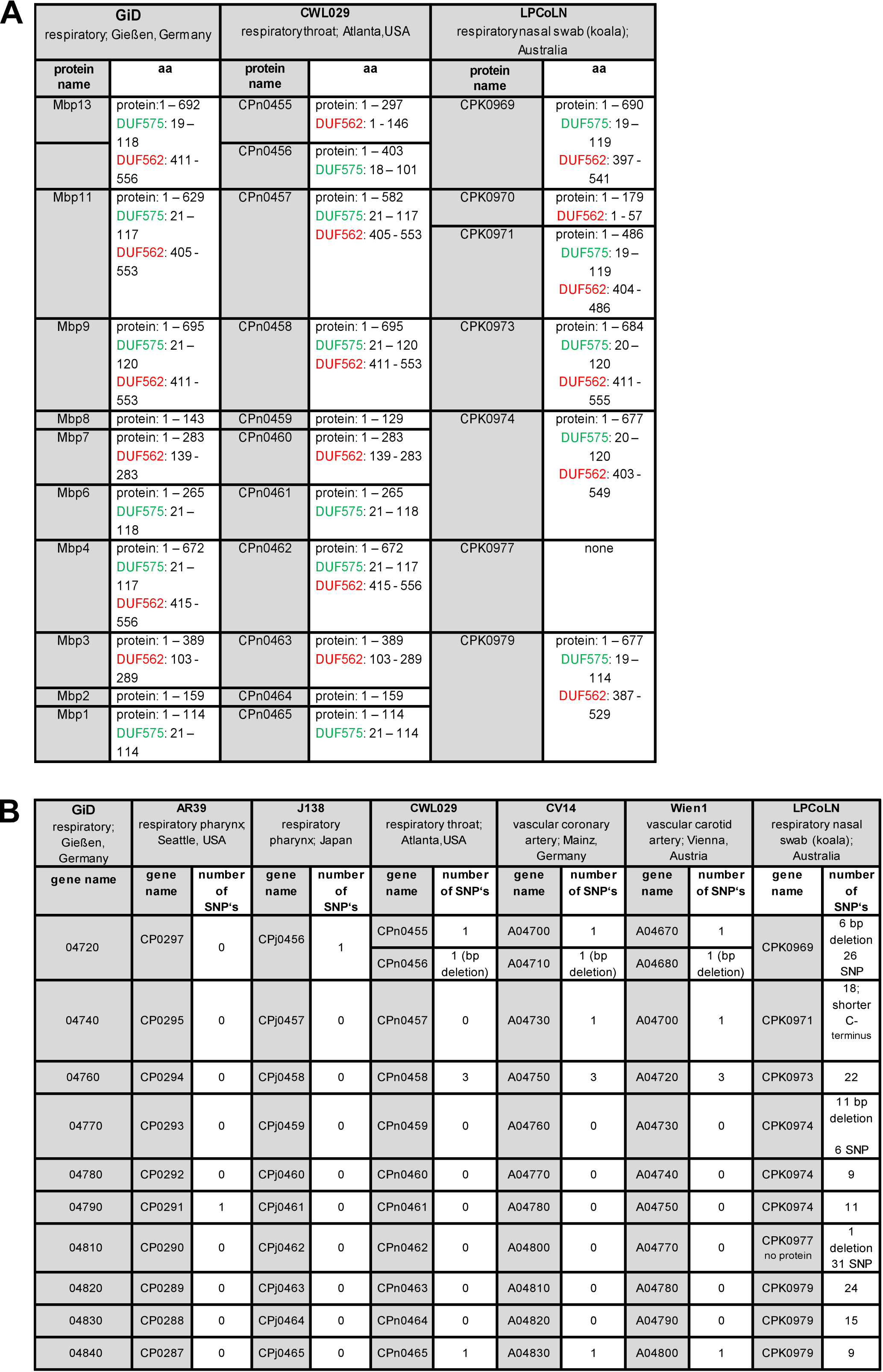

Supplement: Supplementary file 2 [file Image_2.TIF]

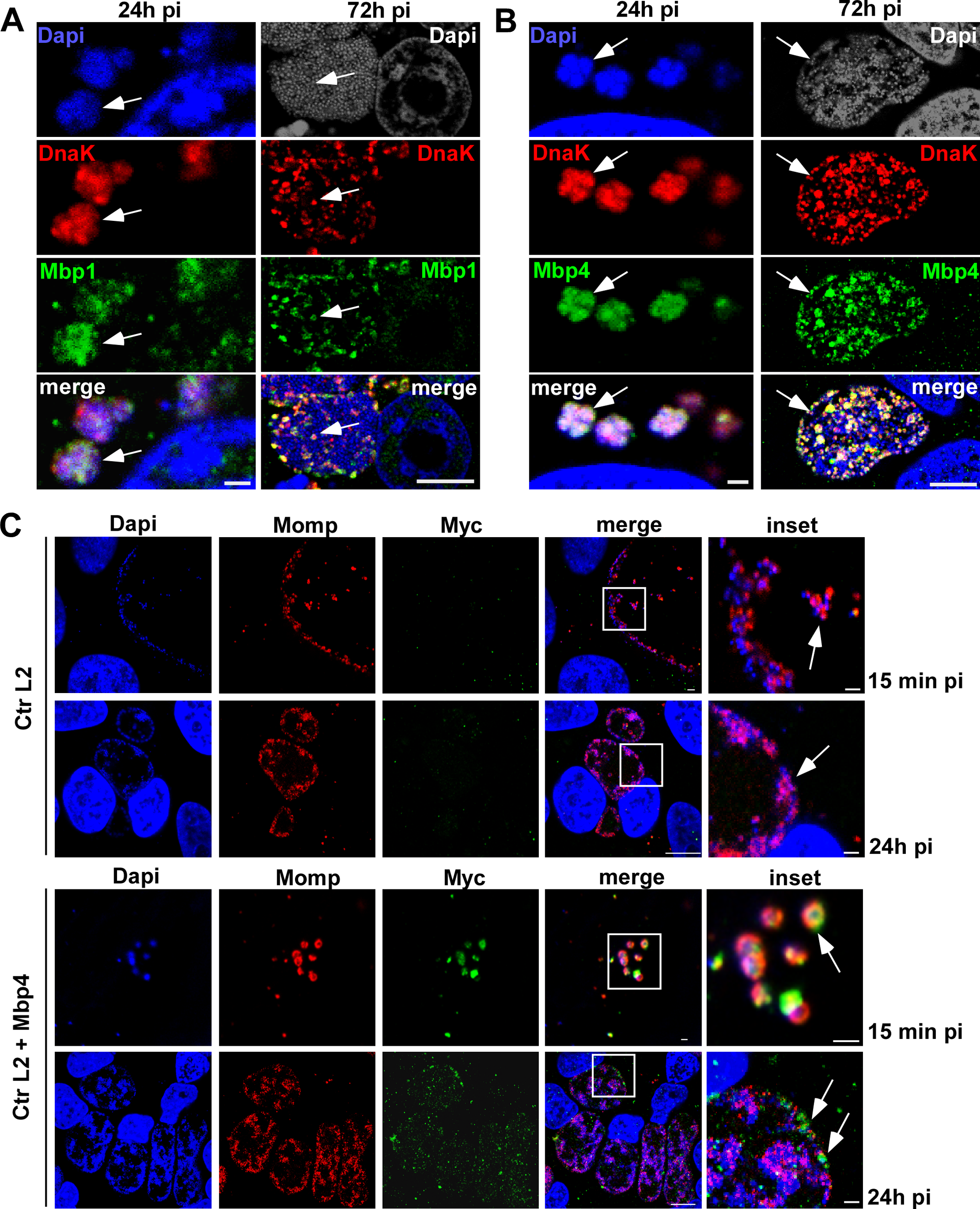

Supplement: Supplementary file 3 [file Image_3.TIF]
