# Supplementary material for: Insights Into a Chlamydia pneumoniae-Specific Gene Cluster of Membrane Binding Proteins
Source: Front Cell Infect Microbiol. 2020 Oct 21;10:565808. doi: 10.3389/fcimb.2020.565808 (PMC7609445; doi:10.3389/fcimb.2020.565808)
Supplement: Supplementary file 4 [file Table_1.docx]

**Supplementary figure legends**

**Fig. S1: Identification of the *Cpn* specific *mbp* gene cluster by partial identity of Mbp1 to the human GTPase Rab36.**

**(A)** Sequence comparison of the hypothetical *Cpn* GiD Mbp1 protein with the human Rab36 GTPase (Isoform CRA b, NCBI EAW59562.1) carried out with MUSCLE https://www.ebi.ac.uk/Tools/msa/muscle/. ***** marks identical amino acids, **:** marks amino acids with high consensus, **.** marks amino acids with low consensus. **(B)** Sequence comparison of predicted products of the GiD *mbp1-mbp13* gene cluster carried out with MUSCLE. The results are listed as % identity. This table presents pairwise overall identity among the proteins of the Mbp1-Mbp13 cluster. Asterisks mark proteins without either DUF domain. **(C)** Amino acid sequence comparison of DUF575 domains of GiD Mbps depicted as alignment (top) and given as table with pairwise identity in % (bottom). **(D)** Alignment of DUF562 domains sequences (top) and table with pairwise identity in % (bottom). The blue box marks the highest, the black box the lowest level of pairwise identity found.

**Fig. S2: Variations in the *mbp1-mbp13* gene cluster among various human *Cpn* isolates and the koala-derived *Cpn* strain LPCoLN.**

**(A)** Size and localization of DUF575/ DUF562 domains within cluster proteins of GiD, CWL029 and koala strain LPCoLN. DUF575 and DUF562 residues are indicated in green and red, respectively. **(B)** Comparisons of SNP and IN/DEL distribution within the ten large genes of the cluster in six different *Cpn* isolates including the koala isolate LPCoLN, relative to the reference isolate GiD.

**Fig. S3: Proteins of the Mbp cluster associate with outer membrane protein Momp and are not T3 secreted.**

**(A, B)** Confocal images of *Cpn* infected cells fixed at 24h and 72 hpi and stained for DnaK visualized with anti-mouse Alexa594 and specific antibodies against Mbp1 **(A)** or Mbp4 **(B)** both visualized with anti-rabbit Alexa488. DNA was stained with Dapi. White arrows indicate colocalization of Mbp1/Mbp4 with DnaK at 24 and 72 hpi. Bar 1µm (24 hpi). Bar 10µm (72 hpi). The images are representative for 3 different biological replicates (n=3). **(C)** Confocal images of cells infected with either wildtype *Ctr* L2 or transformed *Ctr* L2 expressing Mbp4-2xMyc from a plasmid. Cells were infected for 15 min with purified EBs from both strains at a MOI= 10 or for 24 h with an MOI= 1. At indicated time points cells were fixed and stained with anti-Myc and anti-Momp antibody visualized by anti-rat Alexa488 and anti-mouse Alexa594. DNA was stained with Dapi. White boxes are shown in insets, white arrows indicate colocalization of Mbp4 with Momp. Bar 1µm (15 min pi and insets). Bar 10µm (24 hpi). The images are representative for 2 different biological replicates (n=2).
